# The effects of retinal disease on intrinsic protein disorder and liquid–liquid‑phase separation

**DOI:** 10.1007/s42485-025-00188-6

**Published:** 2025-06-19

**Authors:** Nedym Hadzijahic, Colin K. Kim, Mak B. Djulbegovic, Michael Antonietti, David J. Taylor Gonzalez, Vladimir N. Uversky, Jose S. Pulido, Carol L. Karp

**Affiliations:** 1 Bascom Palmer Eye Institute, University of Miami, 900 NW 17th Street, Miami, FL 33136, USA; 2 Wills Eye Hospital, Thomas Jefferson University, Philadelphia, PA, USA; 3 Hamilton Eye Institute, University of Tennessee Science Center, Memphis, TN, USA; 4 Department of Molecular Medicine and USF Health Byrd Alzheimer’s Research Institute, Morsani College of Medicine, University of South Florida, Tampa, FL, USA; 5 Department of Ophthalmology, Massachusetts Eye and Ear Infirmary, Harvard Medical School, Boston, MA, USA; 6 Translational Ophthalmology, Wills Eye Hospital, Philadelphia, PA, USA

**Keywords:** Retina proteome, Intrinsic disorder, Liquid–liquid-phase separation, Age-related macular degeneration, Inherited retinal diseases, Glaucoma, Diabetic retinopathy

## Abstract

**Background:**

The human retina is integral to vision, converting light into neural signals through a complex interplay of specialized neuronal cell types. Recent proteomic studies have revealed significant insights into retinal function, yet much of the retina’s proteome remains unexplored. Our research focuses on quantifying and characterizing intrinsically disordered proteins (IDPs) and regions (IDRs) within the retina and other ocular structures. These proteins are critical for cellular processes due to their flexible, structure-less nature, allowing for versatile interactions in signaling and regulatory networks. Furthermore, we investigate the phenomenon of liquid–liquid-phase separation (LLPS), a process vital for cellular organization and implicated in various diseases, within the retina proteome.

**Methods:**

In this study, we employed a suite of bioinformatics and deep learning tools to analyze protein intrinsic disorder and the propensity for LLPS in proteomes from both healthy and diseased retinas. We utilized the Human Protein Atlas (HPA) as a baseline control, comparing it against the RetNet protein set and samples afflicted by age-related macular degeneration (AMD), glaucoma, and diabetic retinopathy (DR) with and without gliosis. Protein sequences were sourced from the universal protein resource (UniProt) and analyzed for intrinsic disorder using the rapid intrinsic disorder analysis online (RIDAO) platform. Disorder levels and phase separation tendencies were further examined through statistical analyses, including ANOVA and chi-squared tests, to evaluate differences across proteomes. In addition, we assessed the likelihood of proteins to undergo LLPS using predictive tools, such as PSPredictor and ParSe V2, integrating these findings with intrinsic disorder data to draw comprehensive conclusions about the structural dynamics within these proteomes.

**Results:**

The HPA control proteome displayed the highest levels of intrinsic disorder, significantly greater than those observed in disease-specific proteomes, including those affected by AMD, glaucoma, and diabetic retinopathy with and without gliosis. CH–CDF plot analysis revealed distinct structural profiles, with a higher proportion of structured proteins in the HPA and molten globular states prevalent in disease states. Our findings highlight a marked disparity in LLPS propensity, with the HPA proteome and the RetNet Protein Set demonstrating the greatest potential, suggesting a disease-specific alteration in protein interaction dynamics and structural organization.

**Discussion:**

This study revealed significant variations in protein intrinsic disorder and liquid–LLPS across healthy and diseased retinal proteomes. The highest levels of disorder in the HPA proteome suggest a proteomic flexibility that is critical for normal retinal function. In contrast, the AMD and glaucoma proteomes, with their lower disorder and LLPS propensity, may lack this adaptability, potentially contributing to disease progression. These insights underscore the importance of protein dynamics in retinal disorders and point towards targeted therapies that could manipulate these properties to improve or maintain retinal health.

## Introduction

The human retina is a highly specialized tissue located at the back of the eye, responsible for converting light into neural signals that the brain translates into vision (Nguyen et al. 2024; Hoon et al. 2014). This thin layer of tissue is composed of several neuronal cell types, including photoreceptors, horizontal cells, bipolar cells, amacrine cells, and ganglion cells, each contributing to the processing of visual information (Nguyen et al. 2024; Hoon et al. 2014; Yan et al. 2020). The interplay of these various retinal cell types is vital for the seamless transmission of visual information from the eye to the brain (Hoon et al. 2014; Demb and Singer 2015; Gilbert 2015). In recent years, the study of proteins within the retina has unveiled intriguing insights into the mechanisms underlying retinal function (Tsin et al. 2018). However, there remain unexplored areas of the proteome, offering potential for discoveries in retinal biology.

Our group has recently started to quantify and characterize protein intrinsic disorder in other ocular structures, including the aqueous humor, vitreous body, and tears (Djulbegovic and Uversky 2022; Taylor Gonzalez et al. 2023; Antonietti et al. 2024). Intrinsically disordered proteins (IDPs) or proteins with intrinsically disordered regions (IDRs) have become of interest due to their versatile role in a variety of cell processes (Wright and Dyson 2015; Bondos et al. 2022; Iakoucheva et al. 2002; Tantos et al. 2012; Bürgi et al. 2016). IDPs/IDRs lack a fixed or ordered three-dimensional structure under physiological conditions, enabling them to participate in complex signaling and regulatory networks essential for retinal development and function (Chakrabarti and Chakravarty 2022; Uversky 2013). Proteins that exhibit a high degree of intrinsic structural disorder are flexible and versatile and can interact with multiple partners, which facilitates their ability to take part in a large range of cellular functions. (Trivedi and Nagarajaram 2022; Zanotti 2023; Shirai and Kikuchi 2013; Li et al. 2012)

IDPs and proteins with IDRs are also prone to undergoing liquid–liquid-phase separation (LLPS) (Brocca et al. 2020a; Guseva et al. 2023; Tong et al. 2022). LLPS, an emerging field in cellular biology, describes the formation of membrane-less organelles that play key roles in gene regulation (Hirose et al. 2023; Dai and Yang 2024; Tong et al. 2022). These processes have gained attention as cellular mechanisms that contribute to transcription, chromatin organization, and DNA damage response (Brocca et al. 2020a; Guseva et al. 2023; Peng et al. 2021). LLPS is crucial for cellular compartmentalization, allowing for the spatial and temporal regulation of biological reactions without the need for membrane-bound structures (Brocca et al. 2020a; Guseva et al. 2023; Peng et al. 2021; Zhang et al. 2022; Zhou et al. 2022). Recent research highlights the importance of LLPS in the development and progression of various diseases, including neurodegenerative disorders and cancers, thus underscoring its potential as a therapeutic target (Tong et al. 2022; Liu et al. 2023). Understanding the mechanisms underlying LLPS can provide new insights into cellular function and disease pathology.

To our knowledge, no previous research has explored the relationship between protein intrinsic disorder and the propensity of proteins to undergo LLPS in healthy and diseased retina proteomes. Our comprehensive study aims to delineate the relationship between various retinal diseases and their impact on protein intrinsic disorder and the tendency for LLPS within the retina proteome. Using data from established retinal databases, such as the human protein atlas (HPA) and RetNet, along with proteomic data from experimental studies of retinas affected by diseases, including age-related macular degeneration (AMD), glaucoma, and diabetic retinopathy with and without gliosis, we aim to uncover how disease states modify proteomic structures and functionalities. We hypothesize that there will be significant differences in the levels of protein intrinsic disorder among retinas affected by different disease states compared to healthy controls. These variances in intrinsic disorder are expected to correlate with various potentials for LLPS, suggesting a disease-specific alteration in protein interaction dynamics and cellular compartmentalization mechanisms. Through our investigation, we offer insights into how levels of intrinsic disorder and the tendency to undergo LLPS in the retina proteome influence the development and progression of these conditions.

## Methods

In this study, we use a suite of deep learning and bioinformatics tools to conduct a comparative analysis of protein intrinsic disorder and propensity for LLPS among proteins within the proteomes of both healthy and diseased retinas, aiming to understand the impact of IDPs on retinal function. Our control group utilizes the defined retinal proteome from the Human Protein Atlas (HPA), a Swedish-based open-access repository started in 2003 (The Human Protein Atlas 2025; Thul and Lindskog 2018). This proteome serves as a healthy baseline for comparison with five other retinal proteomes, each reflecting a different disease state of the retina. One group is a protein set derived from RetNet, a database of over 280 genes implicated in 27 different inherited retinal diseases (IRDs), including Bardet–Biedl syndrome, retinitis pigmentosa, and Usher syndrome (RetNet 2024). The four other proteomes were derived from retinas affected by: age-related macular degeneration (AMD), glaucoma, diabetic retinopathy (DR) with gliosis, and DR without gliosis (Funke et al. 2016; Sundstrom et al. 2018; Zhang et al. 2015). Gliosis describes a non-neoplastic proliferation of retinal glial cells in response to retinal injury (Yanoff et al. 1971; Jakobiec et al. 2016). These five proteomes reflecting different retinal pathologies were compared to the healthy control retinal proteome to better understand the role that intrinsic disorder and protein phase separation have in the retina.

### Proteome protein identification

Using the genes identified in the HPA (control) proteome and the diseased (experimental or comparison), we obtained canonical primary amino acid sequences from the Universal Protein Resource (https://www.uniprot.org/, accessed on 5/15/24) (Bairoch et al. 2005; Consortium 2022). Starting with the identified genes, the ID mapping tool was used to return a list of unique UniProt IDs for each proteomic group. To ensure scientific accuracy, only matching UniProt IDs from the Swiss-Prot section of the UniProt KnwoledgeBase (KB) were used. Swiss-Prot was created in 1986 and is a manually maintained and reviewed protein sequence database, using experimental results and computed features to form a reviewed and verified section of the entire UniProtKB (Bairoch and Apweiler 2000). From the unique UniProt IDs, FASTA sequences were obtained to further analyze the proteins in each of the six proteomes. Importantly, not all genes identified in the six proteomes could be mapped to a UniProt ID from Swiss-Prot, thus had no canonical primary amino acid sequences and were not used in downstream analysis. These FASTA files for each of the six proteomes were used for all downstream analyses and are available in the supplementary files as Supplementary File X.

### Human protein atlas (HPA) proteome

We derived our control group Human Protein Atlas (HPA) proteome from the retinal transcriptome available on the HPA, an open-access repository (available at https://www.proteinatlas.org/humanproteome/tissue/retina, accessed on 5/15/24) (The Human Protein Atlas 2025). The transcriptome was obtained through antibody-based protein profiling, utilizing immunohistochemistry to detect protein expression in various cell types within the retina (Thul and Lindskog 2018). There were 13,120 proteins found to be expressed in the human retina. (The Human Protein Atlas 2025)

### Diseased retinal proteomes

Our RetNet Protein Set came from RetNet (available at https://web.sph.uth.edu/RetNet/, accessed on 5/18/24), a website that provides over 280 genes implicated in 27 different IRDs (Daiger et al. 2024). All the genes listed on RetNet were collected and used for protein identification. This resulted in 331 proteins listed on RetNet that were mapped with UniProt IDs.

Our AMD proteome was derived from a study by Zhang et al. In their study, protein extraction involved dissection of retina from the retinal pigment epithelium/choroid and vitreous of 5 human donor eyes (aged 51–76 years) (Zhang et al. 2015). They isolated 158 proteins associated with AMD, which were used for downstream analysis (Zhang et al. 2015).

Our glaucoma proteome was derived from a study by Funke et al., which analyzed 5 human retina samples affected by glaucoma and 5 samples not affected by glaucoma (Funke et al. 2016). Retina tissue of donors was extracted from pigment epithelium and isolated in phosphate buffered saline (Funke et al. 2016). They utilized high-performance mass spectrometry to identify over 600 proteins across all samples, isolating 32 proteins that were either only detected in the glaucoma samples or were distinctly elevated in the glaucoma samples (Funke et al. 2016).

Our diabetic retinopathy (DR) proteome was derived from a study by Sundstrom et al., which analyzed 10 different retinas (Sundstrom et al. 2018). They analyzed 5 postmortem retinas without glial activation and five retinas with glial activation, identifying 2,567 proteins across both groups (Sundstrom et al. 2018). We analyzed these as two independent groups based on the presence of glial activation. The DR proteome with gliosis was labeled “DR( +)G” and the DR proteome without gliosis was labeled “DR(−)G.” These two proteomes were considered as independent proteomes for all downstream analysis.

### Analysis of intrinsic disorder within the proteomes

#### Prediction of disorder using commonly used predictors

In the next portion of this study, we quantified intrinsic disorder at the level of individual residues using the Rapid Intrinsic Disorder Analysis Online (RIDAO) platform (available at (https://RIDAO.app) (Dayhoff and Uversky 2022). This platform employs various per-amino acid residue predictors of intrinsic disorder, including the predictors of naturally disordered regions (PONDR)® VSL2, VL3, VLXT, and FIT, along with IUPred short and long to calculate the average disorder scores (ADS) and percentages of predicted disordered residues (PPDR). ADS quantifies the average disorder for a given protein, while PPDR measures the proportion of amino acids within a protein that possess a predicted disorder score above 0.5.

Following the determination of protein intrinsic disorder at the individual amino acid level using RIDAO predictors, we performed analysis of variance (ANOVA) tests to test for statistical significance of variations in ADS and PPDR across our protein groups. This analysis allowed us to determine whether the differences in intrinsic disorder among the various retinal proteomes was statistically significant or merely due to chance. This provided a statistical framework for our analysis to better understand the relationship of intrinsic disorder between the proteomes.

#### Average disorder vs percent of predicted disorder residues analysis

Based on an established technique from our previous papers, we continued our per-residue analysis using data from PONDR® VSL2, which was retrieved from the RIDAO output (Dayhoff and Uversky 2022; Peng et al. 2005). PONDR VSL2 is designed to assess each amino acid for intrinsic disorder, and its efficacy has been previously shown in the Critical Assessment of Protein Intrinsic Disorder (Necci et al. 2021). Proteins were characterized as highly ordered if their percentage of predicted disordered residues (PPDR) was less than 10% or their Average Disorder Score (ADS) was below 0.15. Moderately disordered proteins were those with 10% ≤ PPDR < 30% or 0.15 ≤ ADS < 0.5. Proteins were deemed highly disordered if they had PPDR ≥ 30% and ADS ≥ 0.5. These same thresholds were used in our previous studies that explored intrinsic disorder in other parts of the eye (Djulbegovic and Uversky 2022; Taylor Gonzalez et al. 2023; Antonietti et al. 2024). This classification allowed us to delineate the proteins into distinct categories based on their intrinsic disorder, facilitating a nuanced structural analysis.

The ADS and PPDR metrics provided complementary insights: the ADS gave an individual disorder prediction for each protein, whereas the PPDR indicated the proportion of amino acids within a protein predicted to be disordered. It’s crucial to recognize that ADS is not directly proportional to PPDR, which led us also to evaluate proteins on ADS alone, categorizing them as highly ordered (ADS < 0.15), moderately disordered (0.15 ≤ ADS < 0.5), and highly disordered (ADS ≥ 0.5).

### CH–CDF plot analysis

The CH–CDF plot analysis was conducted to evaluate intrinsic disorder at the protein level. This involved using the charge-hydropathy (CH) plot and the cumulative distribution function (CDF) to integrate a protein’s net charge and hydrophobicity with the frequency distribution of the predicted disorder. The CH plot can predict a protein’s disorder level by combining its net charge and hydrophobicity. Meanwhile, the CDF can evaluate the frequency of distribution along an entire protein. These two tools have different methods of evaluating intrinsic disorder, so combining them gives us useful information to assess the proteomes. This approach stratified proteins into distinct structural categories, allowing for a versatile analysis of their structural characteristics.

These tools collectively allowed us to categorize proteins within a two-dimensional space, identifying trends in structural properties ranging from ordered to various levels of disorder (Xue et al. 2009; Huang et al. 2012). For each quadrant of the CH–CDF plot, we identified proteins likely structured, disordered, or with mixed characteristics, providing a clear distinction between structured and unstructured proteins. This method, also derived from RIDAO outputs (available at (https://RIDAO.app), simplifies the interpretation of a protein’s overall disorder status.

Each quadrant of the CH–CDF plot provides insight into the likely structural state of proteins: Quadrant 1 (Q1, Bottom Right) includes proteins with a negative CH score and a positive CDF score, indicative of structured proteins. Quadrant 2 (Q2, Bottom Left) contains proteins that may be in a molten globular state or exhibit hybrid characteristics, as both CH and CDF scores are negative, suggesting a lack of unique tertiary structure. Quadrant 3 (Q3, Top Left) encompasses highly disordered proteins with positive CH scores and negative CDF values. Quadrant 4 (Q4, Top Right) includes proteins that appear disordered based on the CH plot but ordered according to the CDF plot. This classification system enhances our analysis by providing a flexible understanding of the range of structural states, from ordered to various levels of disorder, within our proteomes.

To conduct an analysis of protein distribution across the four quadrants in the CH–CDF plot, we again employed a χ^2^ test. By comparing the expected frequencies of protein distribution across the four quadrants with the actual distribution, the *χ*^2^ test determines whether the distributions of proteins across the four quadrants are due to random chance or are statistically significant. Once again, the DOF = (r – 1) × (c – 1), where the c represents columns and r represents rows. The six rows are the different proteomes and protein sets: HPA (control), RetNet, AMD, glaucoma, DR( +) G, and DR(−)G. The columns are the four distinct quadrants: Q1, Q2, Q3, and Q4. Thus, the product of the differences in the calculation provided a final DOF value of 15.

### Likelihood for each proteome to undergo liquid–liquid‑phase separation (LLPS)

To investigate the propensity for liquid–liquid-phase separation (LLPS) among proteins in this study, we utilized two bioinformatics tools: the PSPredictor tool (Chu et al. 2022) (available at http://www.pkumdl.cn:8000/PSPredictor/, accessed on 5/22/24) and ParSe V2 (Wilson et al. 2023) (Partition Sequence, available at https://stevewhitten.github.io/Parse_v2_web/, accessed on 5/25/24). The PSPredictor tool is a sequence-based machine learning model developed to predict phase separating proteins (PSPs). This tool was chosen based on its demonstrated high accuracy and ability to generalize across diverse protein types without reliance on specific protein features. The PSPredictor tool, detailed by Chu et al. (2022), combines componential and sequential information during the protein embedding stage and applies a Gradient Boosting Decision Tree (GBDT) algorithm to predict the likelihood of proteins undergoing LLPS (Chu et al. 2022). The PSPredictor achieves a robust tenfold cross-validation accuracy of 94.71% and has been shown to outperform several first-generation PSP prediction tools. This high level of precision is particularly beneficial for identifying potential scaffolds and clients involved in the formation of membraneless organelles, which are crucial for various cellular functions and have been linked to several diseases when dysregulated. (Chu et al. 2022)

ParSe V2 was utilized to evaluate proteomes for propensity towards LLPS by identifying phase separating intrinsically disordered regions (PS IDRs) within a given protein sequence (Wilson et al. 2023). This predictor is based on the established relationship between the hydrodynamic size of proteins and their propensity to induce phase separation, using the human proteome as a reference. (Lin and Chan 2017; Lin et al. 2020; Dignon et al. 2018)

To evaluate our six proteomes for their corresponding likelihood to undergo LLPS, we inputted each post-UniProt mapped FASTA file into these two predictors. Using the PSPredictor, we categorized proteins into potential PSPs based on their scores, with higher scores indicating a greater propensity for undergoing phase separation. We then integrated these predictions with our broader analysis of intrinsically disordered regions (IDRs) and their potential role in the retina. For ParSe V2, the output was used to chart a line for each of the six proteomes. The *x*-axis of this graph represents the length of a given PS IDR, while the *y*-axis indicates the percentage of proteins in the proteome with a PS IDR at least as long as the length shown on the *x*-axis. For instance, a point at [50 (x-coordinate), 15 (y-coordinate)] would mean that 15% of the proteins in the specified proteome have a PS IDR that is at least 50 amino acids long. One proteome that is known to exhibit LLPS was shown by Vernon et al. to have 90% of its proteins containing PS IDRs ≥ 50 amino acid residues in length (Vernon et al. 2018). Therefore, in our analysis, we considered a proteome with a higher percentage of longer PS IDRs (≥ 50 residues) as more likely to undergo phase separation than with shorter PS IDRs. In addition, recall plots were generated for each of the six proteomes against the reference human proteome used in the design and training of ParSe V2 (Wilson et al. 2023). These recall plots provided an area under the curve (AUC) for each proteome, with an AUC > 0.5 indicating the existence of sequences with greater phase separation potential when compared to the reference human proteome. (Wilson et al. 2023)

### Overlap analysis

To investigate the relationship between disease-specific retinal proteomes and the control HPA proteome, we conducted an analysis comparing the features of proteins unique to each disease proteome with those of proteins overlapping with the HPA proteome. For each proteome (AMD, DR with/without gliosis, Glaucoma) and the RetNet Protein Set, we assessed intrinsic protein disorder and LLPS propensity between the unique proteins and the overlapping proteins. ANOVA was used to determine differences in these features between the unique and overlapping protein groups within each proteome.

## Results

### Proteome protein identification

For each of the six groups (i.e., HPA control proteome and 5 disease proteomes), UniProtKB/SwissProt was used to identify all the proteins and define our six proteomes. After post-UniProt mapped FASTA files were obtained, there were 12,844 proteins identified in the human protein atlas (HPA) proteome, 331 proteins identified in the RetNet protein set, 158 proteins identified in the age-related macular degeneration (AMD) proteome, 174 proteins identified in the diabetic retinopathy with gliosis (DR( +)G) proteome, and 104 proteins identified in the diabetic retinopathy without gliosis (DR(−)G) proteome. It is important to note that the numbers of proteins in the final proteomes were fewer than the numbers of proteins identified in the initial parent studies, as utilization of SwissProt/UniProtKB only returned protein entries that were reviewed and verified. This limited the number of UniProt ID matches but ensured accuracy of the downstream FASTA analysis.

### Analysis of intrinsic disorder within the proteomes

#### Prediction of disorder using commonly used predictors

The subsequent aim of our research was to measure and contrast the protein intrinsic disorder tendencies at the amino acid level among the six categorized retinal proteomes. These proteomes included the HPA retinal proteome, RetNet protein set, AMD proteome, glaucoma proteome, DR(−) G proteome, and DR( +)G proteome.

The RIDAO tool provided two separate metrics of intrinsic disorder: the percentages of predicted disordered residues (PPDR) and Average Disorder Scores (ADS) for each group (Table 1). Our analysis was focused on outputs of the PONDR® VSL2B predictor, which has been shown to be effective in predicting intrinsic disorder and is consistent with our previous work. (Djulbegovic and Uversky 2022; Antonietti et al. 2024; Necci et al. 2021; Gonzalez et al. 2023)

The PPDR values for the retinal proteomes ranged from 33.260 to 43.660%, with the ADS values spanning from 0.425 to 0.482. The HPA Proteome exhibited the highest PPDR at 43.660% and an ADS of 0.482. The RetNet protein set had a PPDR of 40.382% and an ADS of 0.462. The AMD proteome had a PPDR value of 33.260% and an ADS value of 0.425. The glaucoma proteome showed a PPDR value of 33.382% and an ADS value of 0.427. Both DR proteomes showed similar disorder levels, with the DR(−)G proteome having a PPDR of 35.768% and an ADS of 0.469, and the DR( +)G proteome having a PPDR of 33.827% and an ADS of 0.468.

Statistical analysis using ANOVA revealed significant differences in both PPDR and ADS among the six groups (ANOVA F-statistic = 13.626 and 8.827, respectively, *p* values < 0.0001 for both). Pairwise *T* test analysis for RIDAO-based PPDR and ADS outputs of the other five disorder predictors showed similar significant differences among the six groups (Supplemental Table 1).

#### Average disorder score vs percent of predicted disorder residues analysis

Next, we assessed the distribution of protein disorder classifications across the six retinal proteomes (Table 2). The HPA proteome had 7970 proteins (62.17%) classified as highly disordered (ADS > 0.5 or PPDR > 30%). The RetNet Protein Set had 187 proteins (56.50%) categorized as highly disordered. In the AMD proteome, 70 proteins (44.30%) were classified as highly disordered. The Glaucoma proteome had 14 proteins (43.75%) in this category. The DR(−)G proteome had 77 proteins (44.77%) classified as highly disordered. The DR( +)G proteome had a moderate proportion of proteins (48 (46.60%)) that were highly disordered.

Moderately disordered proteins (0.15 < ADS ≤ 0.5 and 10% < PPDR ≤ 30%) constituted a substantial portion of these proteomes. The HPA proteome had 4371 proteins (34.10%) classified as moderately disordered. The RetNet Protein Set had 125 proteins (37.76%). In the AMD proteome, 76 proteins (48.10%) were categorized as such. The Glaucoma proteome had 17 proteins (53.12%). The DR(−) G proteome had 88 proteins (51.16%), while the DR( +)G proteome had 55 proteins (53.40%).

Of the six proteomes, the HPA proteome was the only group that contained highly ordered proteins (ADS ≤ 0.15 and PPDR ≤ 10%), with only 19 proteins (0.15%).

#### CH–CDF plot analysis

Using the CH–CDF analysis, we categorized proteins in each of the six retinal proteomes into four quadrants according to their predicted disorder characteristics. Once again, Quadrant 1 (Q1, Bottom Right) proteins had a negative CH score and a positive CDF score, indicative of high levels of structure. Quadrant 2 (Q2, Bottom Left) contains proteins that may be in a molten globular state or exhibit hybrid characteristics, as both CH and CDF scores are negative, suggesting a lack of unique tertiary structure. Quadrant 3 (Q3, Top Left) proteins are highly disordered with positive CH scores and negative CDF values. Quadrant 4 (Q4, Top Right) includes proteins that appear disordered based on the CH plot but ordered according to the CDF plot. A total of 13,640 proteins across the six proteomes were plotted.

**Fig. 1:**
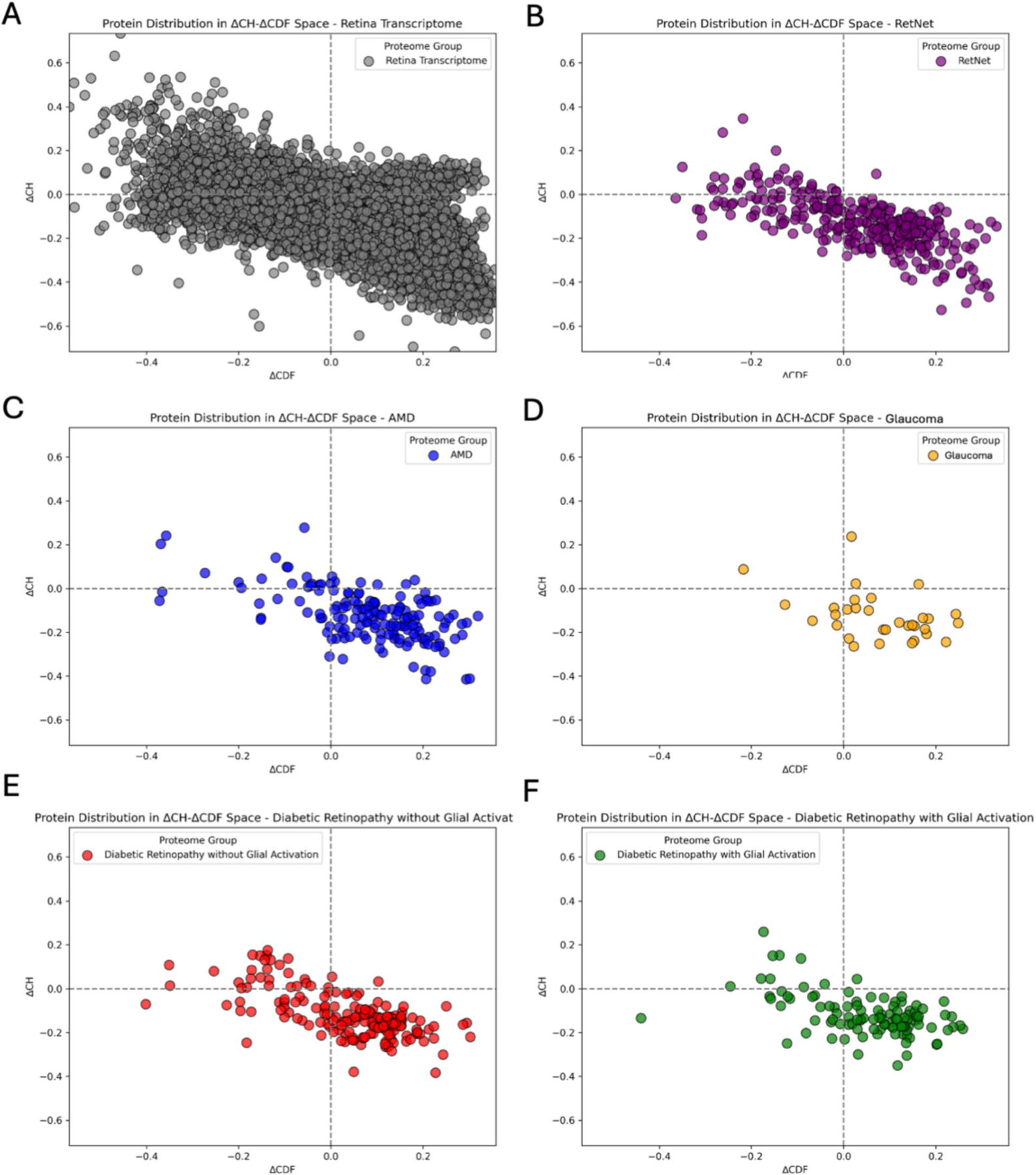
CH (charge hydropathy) and CDF (cumulative distribution function) plots for each of our six proteomes. **A** Human Protein Atlas (HPA) retinal transcriptome. **B** RetNet Protein Set. **C** Age-related macular degeneration (AMD) proteome. **D** Glaucoma proteome. **E** Diabetic retinopathy (DR) without glial activation proteome (DR(−) G). **F** DR with glial activation proteome (DR( +)G)

For the HPA proteome (Fig. 1A), 12,844 proteins were analyzed: Quadrant 1 (Bottom Right) had 7396 proteins (57.70%) in a structured state. Quadrant 2 (Bottom Left) included 3291 proteins (25.67%) identified as having a molten globular structure. Quadrant 3 (Top Left) encompassed 1,671 proteins (13.04%) associated with a high level of disorder. Quadrant 4 (Top Right) had 461 proteins (3.60%) showing mixed structural characteristics.

For the RetNet Protein Set (Fig. 1B), 331 proteins were analyzed: Quadrant 1 had 209 proteins (63.14%) in a structured state. Quadrant 2 included 82 proteins (24.77%) identified as having a molten globular structure. Quadrant 3 encompassed 39 proteins (11.78%) associated with a high level of disorder. Quadrant 4 had 1 protein (0.30%) showing mixed structural characteristics.

For the AMD proteome (Fig. 1C), 158 proteins were analyzed: Quadrant 1 had 119 proteins (75.32%) in a structured state. Quadrant 2 included 15 proteins (9.49%) with a molten globular structure. Quadrant 3 encompassed 18 proteins (11.39%) associated with a high level of disorder. Quadrant 4 had 6 proteins (3.80%) showing mixed structural characteristics.

For the Glaucoma proteome (Fig. 1D), 32 proteins were analyzed: Quadrant 1 had 23 proteins (71.88%) in a structured state. Quadrant 2 included 5 proteins (15.62%) identified as having a molten globular structure. Quadrant 3 encompassed 1 protein (3.12%) associated with a high level of disorder. Quadrant 4 had 3 proteins (9.38%) showing mixed structural characteristics.

For the DR(−)G proteome (Fig. 1E), 172 proteins were analyzed: Quadrant 1 had 112 proteins (65.12%) in a structured state. Quadrant 2 included 31 proteins (18.02%) with a molten globular structure. Quadrant 3 encompassed 27 proteins (15.70%) associated with a high level of disorder. Quadrant 4 had 2 proteins (1.16%) showing mixed structural characteristics.

For the DR( +)G proteome (Fig. 1F), 103 proteins were analyzed: Quadrant 1 had 73 proteins (70.87%) in a structured state. Quadrant 2 included 20 proteins (19.42%) with a molten globular structure. Quadrant 3 encompassed 9 proteins (8.74%) associated with a high level of disorder. Quadrant 4 had 1 protein (0.97%) showing mixed structural characteristics.

To evaluate the distribution of proteins across the different structural classes in the CH–CDF plot, a χ^2^ analysis was conducted. The results demonstrated a significant discrepancy from the expected distribution in the quadrants (χ^2^ of 61.066, *p* value < 0.0001, 15 DOF). This significant difference indicates that the protein distribution pattern across the quadrants is not due to random variation, suggesting distinctive differences in the structural states of the proteins within the various retinal proteomes compared to what would occur by chance. This analysis indicates that different proteomes exhibit distinct structural characteristics, potentially attributable to their specific biological roles and conditions.

### Likelihood for each proteome to undergo liquid–liquid‑phase separation (LLPS)

Using both the PSPredictor and ParSe v2 tools, we evaluated the propensity for liquid–liquid-phase separation (LLPS) among the five retinal proteomes and the RetNet protein set: HPA, RetNet, AMD, glaucoma, DR(−)G, and DR( +)G. The PSPredictor tool provided scores indicating the likelihood of proteins undergoing LLPS, with higher scores suggesting a greater propensity for phase separation.

**Fig. 2:**
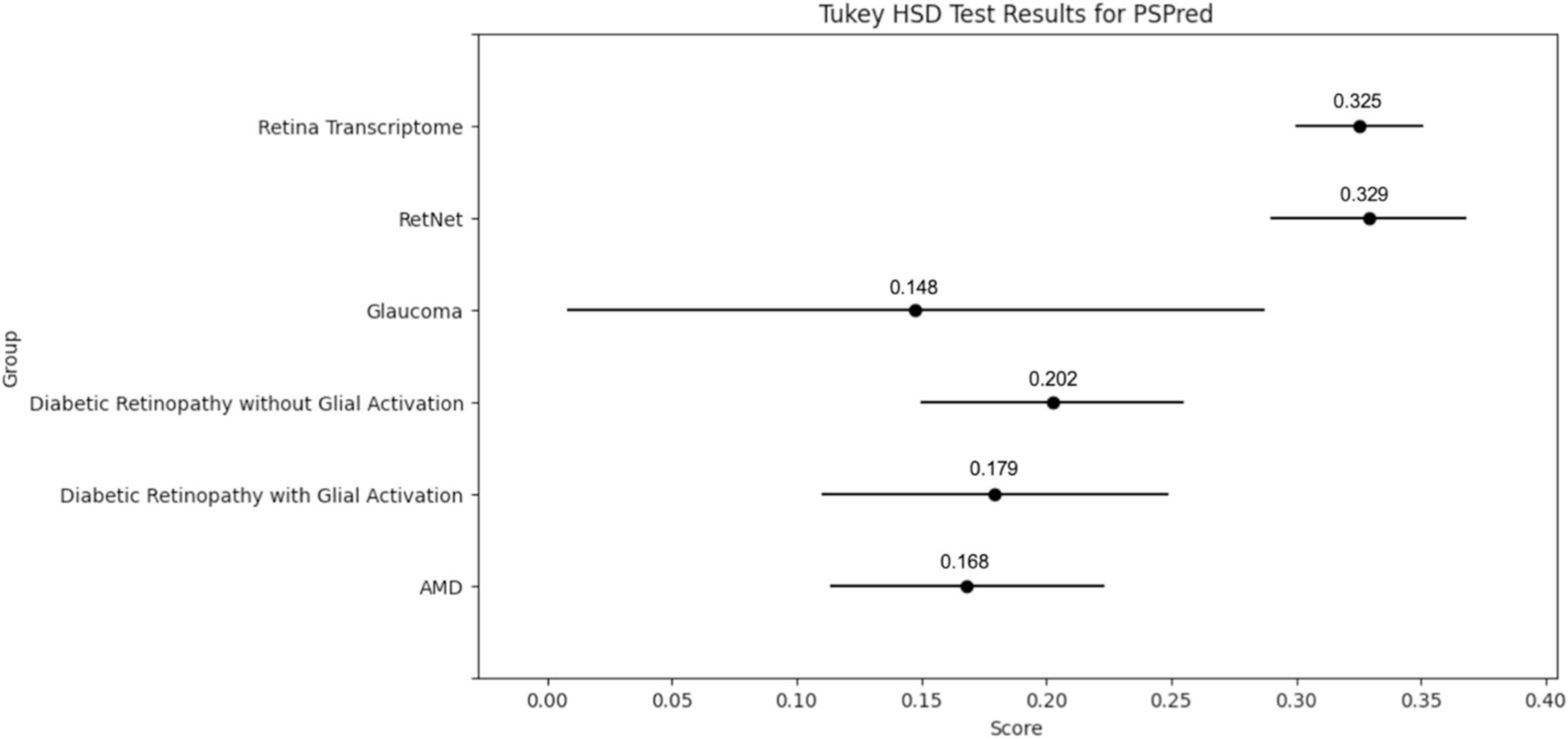
Tukey’s honestly significant difference (HSD) Graph depicting both individual average PSPredictor scores and pairwise differences in average PSPredictor scores. Higher scores indicate a greater likelihood of undergoing liquid–liquid-phase separation (LLPS). RetNet had the greatest score of 0.329, while diabetic retinopathy (DR) with gliosis had the lowest score of 0.032

The average PSPredictor scores (Fig. 2) for each proteome were as follows: HPA (0.325), RetNet (0.329), AMD (0.168), Glaucoma (0.148), DR(−)G (0.202), and DR( +)G (0.179). ANOVA results revealed significant differences in propensity for LLPS among the groups (F-statistic = 15.570, *p* value < 0.0001).

The Tukey honestly significant difference (HSD) test was employed to perform pairwise comparisons of mean PSPred scores between the groups (Fig. 2). AMD showed significant differences when compared to RetNet (*p* < 0.0001) and HPA Retina (*p* < 0.0001). Notably, the HPA proteome and RetNet Protein Set exhibited the highest propensity for LLPS. In contrast, the Glaucoma and AMD proteomes demonstrated the lowest propensities for LLPS.

**Fig. 3:**
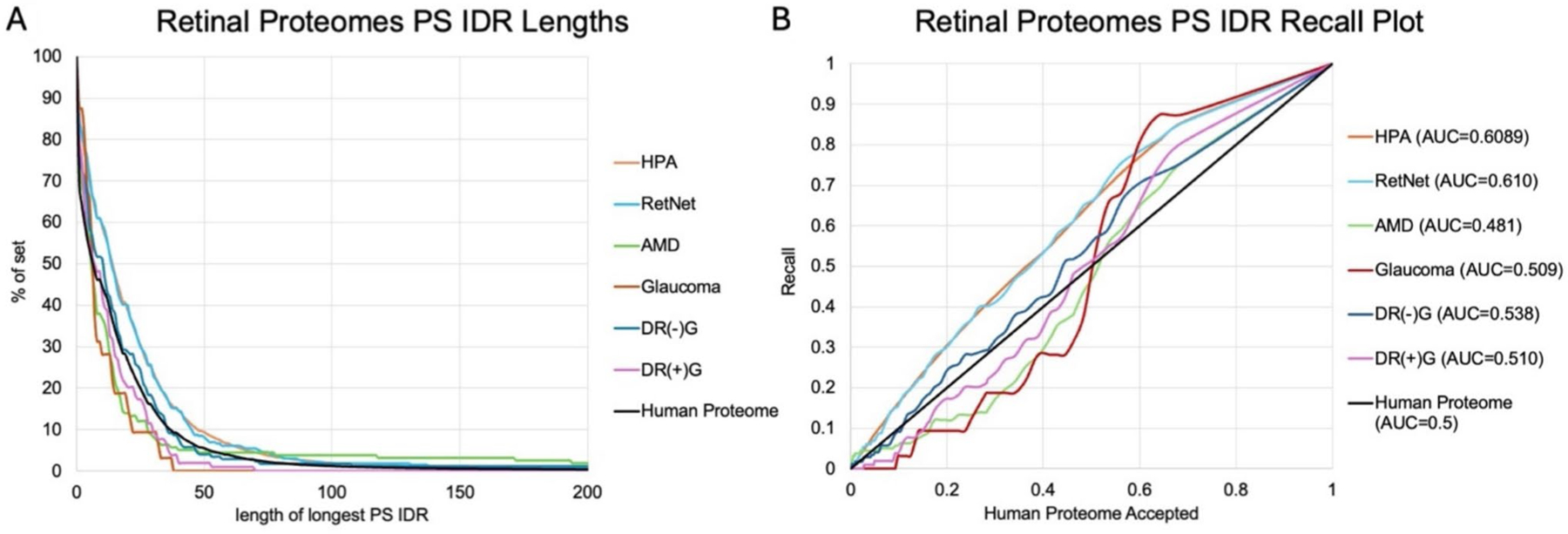
ParSe V2 graphs indicating the likelihood of each subgroup to undergo phase separation. There were six proteomes that were analyzed. Length of identified phase separating intrinsically disordered regions (PS IDRs) were graphed by the percent of the inputted FASTA sequence that had PS IDRs of at least this specific length for the **A** human protein atlas (HPA) retinal proteome, RetNet protein set, age-related macular degeneration (AMD) proteome, diabetic retinopathy (DR) without glial activation proteome (DR(−)G), DR with glial activation proteome (DR( +)G), and ParSe V2 reference human proteome. **B** Recall plots were also generated for these six proteomes. Area under the curve (AUC) of > 0.5 indicated the presence of sequences with greater phase separation potential when compared to the reference human proteome

ParSe V2 was then used to identify phase separating intrinsically disordered regions (PS IDRs) in the six proteomes. PS IDRs were first characterized by their length and the percentage of each proteome that contained PS IDRs of that specified length. These results were graphed for each of the six proteomes (Fig. 3A) alongside the reference human proteome as described by Ibrahim et al. (Wilson et al. 2023; Ibrahim et al. 2023) The HPA proteome and RetNet Protein Set consistently exhibit PS IDRs that are shifted to the right compared to the human proteome control used in ParSe V2, indicating that a higher percentage of their sequences have longer PS IDRs. This trend suggests that these proteomes contain a greater proportion of proteins with significant phase separation potential. In contrast, the AMD, glaucoma, and diabetic retinopathy (DR) proteomes show a distribution more closely aligned with or left-shifted relative to the human proteome, indicating shorter PS IDRs and, potentially, a lower phase separation potential.

Recall plots were also created using ParSe V2 data for the six proteomes, alongside the reference human proteome (Fig. 3B). An AUC > 0.5 denoted a greater propensity of a proteome to undergo LLPS when compared to the reference human proteome (Hirose et al. 2023). The HPA proteome had an AUC of 0.609, and the RetNet Protein Set had an AUC of 0.610. The AMD proteome had an AUC of 0.481, indicating had a similar propensity to undergo protein phase separation than the reference human proteome. The Glaucoma proteome had an AUC of 0.509. DR(−)G had an AUC of 0.538, while DR( +)G had an AUC of 0.510. The ParSe v2 results indicated that both HPA and RetNet have a higher likelihood of undergoing LLPS, which is consistent with our PSPred analysis. Similarly, AMD and DR( +)G were the two proteomes least likely to undergo LLPS, which was once again in agreement with our PSPred analysis.

### Overlap analysis

We compared the features of proteins unique to each retinal disease proteome (AMD, DR with/without glial activation, Glaucoma) and the RetNet Protein Set with those overlapping with the Human Protein Atlas (HPA) control proteome (Table 3). The number of unique proteins identified in each group was as follows: 48 in AMD, 6 in DR with glial activation, 19 in DR without glial activation, 10 in glaucoma, and 41 in the RetNet Protein Set. These were compared to the proteins overlapping with the HPA control group, which included 110 for AMD, 97 for DR with glial activation, 153 for DR without glial activation, 22 for glaucoma, and 290 for the RetNet Protein Set.

After the overlap and unique proteins were identified, we performed statistical analyses to assess differences in intrinsic protein disorder and LLPS propensity across these groups. Using RIDAO-based intrinsic disorder analysis and PSPred-based LLPS propensity analysis, ANOVA revealed significant overall differences between the groups (*p* < 0.05). However, pairwise *T* tests did not show significant differences between the unique and overlapping proteins within each protein group examined (see supplemental figures). For example, proteins unique to AMD compared to AMD proteins overlapping with the HPA did not show statistically significant differences. Tukey’s HSD tests confirmed the lack of significant differences in both intrinsic disorder and LLPS propensity for each comparison made (i.e., unique proteins of each proteome or protein set analyzed versus proteins in each group that overlap with the HPA proteome) (*p* > 0.05).

## Discussion

Our comprehensive bioinformatics study characterized the intricate landscape of protein intrinsic disorder and the propensity for LLPS within the retinal proteome, encompassing both healthy and diseased states. By utilizing deep learning and computational tools, we delved into the comparative analysis of proteins across six distinct proteomes, revealing significant findings that enhance our understanding of retinal biology and its associated disorders. Key results from our investigation include the identification of substantial differences in protein intrinsic disorder and LLPS potential among the retinal proteomes. Notably, the HPA proteome exhibited the highest levels of protein intrinsic disorder, while the proteomes associated with AMD and glaucoma proteomes showed lower levels of disorder. Similarly, the potential for LLPS was highest in the HPA and RetNet protein sets, whereas the glaucoma and AMD proteomes demonstrated the lowest propensity for LLPS. Furthermore, this study highlights variations in the HPA transcriptome and disease states, indicating that these differences in protein intrinsic disorder and LLPS potential provide novel insights into the global proteome changes that occur in these conditions.

Our investigation into the intrinsic disorder of retinal proteomes using the RIDAO platform and the PONDR® VSL2B predictor revealed significant variations in the disorder tendencies among different retinal conditions. The analysis, which included six retinal proteomes, showed that the HPA Retinal Proteome had the highest levels of intrinsic disorder, with a PPDR of 43.66% and an ADS of 0.482. This high degree of intrinsic disorder in the healthy retina highlights the essential role of flexible, disordered proteins in maintaining normal retinal functions and responding to environmental changes. Notably, a similar abundance of intrinsic disorder has been observed in the proteome of the aqueous humor, where 208 proteins were predicted to be highly intrinsically disordered (Djulbegovic and Uversky 2022). This suggests that protein intrinsic disorder is crucial for the functional dynamics of various eye compartments, enabling proteins to engage in diverse interactions and maintain ocular health. (Djulbegovic and Uversky 2022)

The RetNet Protein Set, associated with IRDs, showed moderately high disorder levels (PPDR of 40.38% and ADS of 0.462). This indicates that while structural flexibility is important for coping with genetic mutations, it is not as pronounced as in diabetic retinopathy. The moderately high intrinsic disorder in RetNet Protein Set proteins likely facilitates interactions with a diverse array of molecular partners, helping to mitigate the impact of various genetic mutations (Blaabjerg et al. 2023). Many IRDs are characterized by a high frequency of missense mutations, which intrinsically disordered regions (IDRs) in proteins can tolerate better due to their flexible and adaptable nature. This adaptability allows these proteins to maintain their functionality despite genetic alterations (Lee et al. 2023). Moreover, proteins with high levels of intrinsic disorder are often involved in critical cellular processes, such as signaling, regulation, and molecular recognition, which are essential for retinal cell survival and function in the face of genetic stress. (Lee et al. 2023)

The DR proteomes with and without gliosis [DR( +)G and DR(−)G, respectively], while not as disordered as the control HPA proteome, exhibited low to moderate levels of disorder, with PPDR values around 34–35% and ADS values of 0.430–0.440. The moderate levels disorder in these proteomes suggests that the proteins involved in diabetic retinopathy require both structural flexibility and stability to manage the complex and multifaceted challenges posed by the disease. Structural flexibility likely facilitates dynamic interactions with multiple binding partners, which may be key in responding to the hyperglycemic conditions and oxidative stress characteristic of diabetic retinopathy (Shukla and Tripathy 2024). Another possible explanation for this involves the process of the unfolded protein response (UPR) in diabetic retinopathy. Chronic hyperglycemia can stress the endoplasmic reticulum, leading to activation of the UPR, which can cause cellular dysfunctions in retinal cells (Alam and Akhter 2021). Due to their higher propensity for misfolding, IDPs in the DR proteomes may increase the risk of protein misfolding (Uversky et al. 2008; Uversky 2011). This misfolding can exacerbate the UPR, thereby contributing to the progression of DR (Uversky et al. 2008; Uversky 2011). Prolonged UPR activation can lead to inflammatory responses, vascular changes, and neurodegeneration, thereby contributing to the progression of diabetic retinopathy. Therefore, it is plausible that intrinsic disorder in the conditions of diabetic retinopathy contribute to compensatory mechanisms that preserve retinal function in a diseased state. In addition, our results suggest that ordered proteins are also essential for cellular processes in this condition.

The proteomes for AMD and Glaucoma had the overall lowest levels of intrinsic disorder (PPDR around 33% and ADS around 0.425), suggesting that these diseases may reduce the global flexibility of the retina proteome and may involve more structured proteins. This lower level of disorder might reflect a reliance on more stable and less flexible protein structures to cope with the specific pathological mechanisms of AMD and glaucoma, such as oxidative stress and increased intraocular pressure (Izzotti et al. 2006; Freund et al. 2015). The distinct structural requirements of these diseases underscore the diverse ways in which protein structure and disorder contribute to retinal health and disease.

The CH–CDF plot analysis provided a deeper understanding of the structural characteristics of proteins across the six retinal proteomes. This method allowed us to categorize proteins into four distinct quadrants based on their predicted disorder characteristics: structured, molten globules, highly disordered, and mixed structural characteristics. The HPA Retinal Proteome, representing healthy retina, showed a significant proportion of structured proteins (57.70%), highlighting the necessity of stable protein structures for normal retinal function. In addition, 25.67% of proteins were identified as molten globules, and 13.04% as highly disordered, indicating a balanced presence of flexibility and stability required for the diverse functional demands of the retina. The RetNet Protein Set also demonstrated a high percentage of structured proteins (63.14%), reflecting the importance of structural stability in IRDs. This proteome had fewer highly disordered proteins (11.78%) compared to the healthy retina, suggesting that while some flexibility is necessary, structural integrity is crucial for managing genetic mutations. AMD and Glaucoma proteomes exhibited the highest proportions of structured proteins (75.32% and 71.88%, respectively), with relatively low percentages of highly disordered proteins (11.39% and 3.12%). These findings suggest that the pathophysiology of AMD and glaucoma relies more on structured proteins, possibly due to the nature of the cellular stressors involved in these diseases. (Tezel 2006)

Similarly, the DR proteomes with and without gliosis [DR( +)G and DR(−)G, respectively] were dominated by proteins with a structured state (70.87% and 65.12%, respectively), providing a more definitive statement about the proteomic environment than the RIDAO analysis. This high prevalence of structured characteristics in the DR proteomes suggests a highly rigid environment, where highly structured proteins maintain cellular integrity and function (Bajpai et al. 2016). These proteins may play a role in protecting the cell from the multifactorial stressors of the disease, such as oxidative stress (Bajpai et al. 2016; Kowluru and Chan 2007). Highly structured proteins such as occludin and claudins are crucial for maintaining the integrity of the blood–retinal barrier, preventing fluid leakage and exposure to toxins (Díaz-Coránguez et al. 2017; O’Leary and Campbell 2023). The lower proportion of disordered proteins [8.74% in DR( +)G and 15.70% in DR(−)G] further supports the need for a more structured protein network to manage the disease’s complexity. These findings suggest that disease-specific biological factors drive these structural variations, emphasizing the need for tailored therapeutic approaches. For example, interventions that stabilize protein structures might be more effective for DR, AMD, and Glaucoma.

Our analysis of the propensity for liquid–liquid-phase separation (LLPS) among the six retinal proteomes revealed noteworthy differences, highlighting the potential role of phase separation in retinal diseases. The PSPredictor tool provided average scores indicating the likelihood of proteins undergoing LLPS, with significant variability observed among the proteomes. The RetNet Protein Set and the Retina Transcriptome (HPA) exhibited the highest propensities for LLPS, with average scores of 0.329 and 0.325, respectively. This high propensity suggests that proteins in these proteomes are more likely to form membraneless organelles, which are crucial for various cellular functions, such as signaling, regulation, and stress response (Gomes and Shorter 2019). The presence of these dynamic structures could be essential for managing the complex and multifactorial nature of IRDs and maintaining normal retinal functions.

In contrast, the DR proteomes [both DR( +)G and DR(−) G] had moderate propensities for LLPS, with scores of 0.179 and 0.202, respectively. These scores indicate that while LLPS may play a role in these conditions, it is not as predominant as in the RetNet Protein Set and HPA proteome. This could reflect the different pathological mechanisms involved in diabetic retinopathy, where structured proteins and traditional organelles might be more critical. Interestingly, the AMD and Glaucoma proteomes demonstrated the lowest propensities for LLPS, with average scores of 0.168 and 0.148, respectively. In conjunction with their relatively low levels of intrinsic disorder, these proteomes are less likely to form phase-separated structures. This suggests that the proteins in AMD and Glaucoma might rely more on other mechanisms, such as protein–protein interactions and signaling pathways, rather than forming membrane-less organelles. (Patrick et al. 2020)

Our results suggest that the relationship between intrinsic disorder and LLPS propensity is complex and multifaceted. Intrinsic disorder facilitates the flexibility and interaction diversity required for proteins to engage in various cellular processes (Trivedi and Nagarajaram 2022). Proteins with IDRs are often involved in LLPS due to their ability to undergo dynamic conformational changes and form multivalent interactions (Brocca et al. 2020). This property allows them to contribute to the formation of membraneless compartments in cells, which play significant roles in various cellular functions (Brocca et al. 2020). While intrinsic disorder is a key factor in LLPS, other molecular properties and interactions may also significantly contribute to the phase separation behavior or proteins.

Our analysis comparing unique and overlapping proteins within retinal disease proteomes (AMD, DR with/without gliosis, Glaucoma) and the RetNet Protein Set with the HPA control proteome revealed notable findings. ANOVA indicated significant overall differences between these groups in terms of intrinsic protein disorder and LLPS propensity (*p* < 0.05), suggesting distinct proteomic compositions between the retinal disease proteomes and the HPA control. However, subsequent pairwise *T* tests did not reveal statistically significant differences between the unique and overlapping proteins within each group. This was consistent in both the RIDAO-based intrinsic disorder analysis and PSPred-based LLPS propensity analysis, and was further confirmed by Tukey’s HSD test, which also showed no significant differences when comparing unique proteins to overlapping proteins for each protein set in analyzed (*p* > 0.05). Though the small group sizes may have reduced statistical power, these results suggest that the unique and overlapping proteins within each disease group do not substantially differ in terms of their intrinsic disorder or LLPS propensity.

The overlap in protein composition between disease-specific proteomes and the HPA control suggests that key protein characteristics, such as intrinsic disorder and LLPS potential, remain largely preserved across both physiologic and diseased states. Proteins with high intrinsic disorder and LLPS propensity are known for their functional versatility, including roles in regulation, signaling, and phase separation processes. The lack of significant differences in these features between unique and overlapping proteins indicates that the proteins shared between the diseased and control proteomes retain similar structural and functional roles. While ANOVA showed significant overall differences, the absence of significant findings in pairwise comparisons implies that the proteomic changes observed in retinal diseases may be more subtle. Rather than large-scale structural shifts in protein disorder or phase separation potential, it is possible that disease states reflect nuanced regulatory modifications or post-translational changes that do not drastically alter the proteins’ inherent disorder or LLPS propensity.

These findings highlight the need for further research to uncover more granular differences between unique and overlapping proteins in retinal diseases. Increasing sample sizes, refining analytical techniques, and incorporating experimental validation will be crucial to identifying potential biomarkers or therapeutic targets that could differentiate disease-specific proteins from those shared with healthy tissues. Understanding these distinctions at a deeper level could provide insights into the mechanisms driving ophthalmic diseases and pave the way for new therapeutic strategies.

## Limitations

While this study provides valuable insights into the retinal proteome, it is subject to several limitations. First, our analysis relies heavily on data from the human protein atlas. While this resource is comprehensive, it may not capture the complete spectrum of protein expression in the human body and retina. In addition, any biases or errors in this data set could influence our findings.

Second, the intrinsic disorder predictors used in this study, such as PONDR® VSL2 and IUPred, have inherent limitations. Different predictors may yield slightly different results, and the choice of predictors could influence the conclusions drawn. Moreover, our analysis is computational and bioinformatics-based, lacking experimental validation. Therefore, the predicted disorder characteristics and classifications of proteins should be interpreted with caution and ideally confirmed by experimental studies. For our analysis for propensity to undergo LLPS, ParSe v2 ignores sequences longer than 10,000 residues and shorter than 25 residues. Our HPA protome and DR(−)G both contained sequences greater than 10,000 amino acids, which were not included in the ParSe v2 analysis.

Furthermore, this study primarily focuses on the primary amino acid sequences of proteins and does not account for post-translational modifications, alternative splicing, or other factors that could influence protein structure and function. This could limit the generalizability of our findings. In addition, while the *χ*^*2*^ test provides a measure of the significance of the differences observed, it does not account for multiple testing corrections, which could potentially lead to false positives.

In addition, the CH–CDF plot analysis offers a broad categorization of proteins into structured and disordered categories but does not provide detailed information on the specific types or functions of disorder within these proteins. We also did not account for the expression levels of the identified proteins across different proteomes. Simply identifying these proteins does not provide information about whether they are over- or under-expressed in the respective samples. The absence of quantitative expression data restricts our ability to fully understand the functional relevance of these proteins in various retinal conditions. Future studies should incorporate relative protein expression levels to offer a more comprehensive analysis and insights into their roles.

Finally, there is considerable variability in the proteome collection methods across the different data sets analyzed. The proteins were collected using diverse techniques, potentially leading to inconsistencies in data quality and comparability. In addition, there is a large variation in sample sizes, with some proteomes containing thousands of proteins, while others have only a few hundred, which is likely due to the various experimental techniques used to collect the proteomes. Implementing a uniform, standardized collection method would produce more reliable results by minimizing biases and enhancing comparability across studies. Addressing these issues would significantly improve the robustness of the findings and provide a deeper understanding of the proteins’ roles in various retinal conditions.

Moving forward, experimental validation of our computational predictions and the exploration of how these disordered regions contribute to the molecular mechanisms of retinal diseases could prove indispensable. Moreover, the integration of post-translational modifications and the dynamics of protein–protein interactions in our understanding of intrinsic disorder will likely open new avenues for research, potentially leading to novel diagnostic and therapeutic approaches for diseases linked to the retina and beyond.

## Conclusions

This study provides novel insights into the differential protein intrinsic disorder and LLPS potential across various retinal proteomes, highlighting significant variations between healthy and diseased states. These findings enhance our understanding of the proteome features in retinal health and disease, offering novel perspectives on the global changes occurring in these proteomes.

## Supplementary Material

S3

S2

S4

S5

S6

Supplemental 1

The online version contains supplementary material available at https://doi.org/10.1007/s42485-025-00188-6.

## Figures and Tables

**Table 1:** Number of proteins within each of the six analyzed proteomes, along with their average disorder scores (ADS) and percentages of predicted disordered residues (PPDR) using the output of PONDR® VSL2B and the statistical differences among the six groups

Group Analyzed	Number of Proteins in Proteome	PPDR (VSL2B), %	ADS (VSL2B)
Human Protein Atlas (HPA) Control Retinal Proteome	12,844	43.660	0.482
Disease Retinal Proteomes			
RetNet Protein Set	331	40.382	0.462
Age-related macular degeneration (AMD) proteome	158	33.260	0.425
Glaucoma proteome	32	33.382	0.427
Diabetic retinopathy (DR) without gliosis (DR(−)G) proteome	172	35.768	0.440
Diabetic retinopathy with gliosis (DR( +)G) proteome	103	33.827	0.430
ANOVA F-statistic		13.626	8.827
ANOVA *p* value		< 0.0001	< 0.0001

**Table 2:** Average disorder (ADS) vs percent of predicted disorder residues (PPDR) analysis

	Highly Disordered, number of proteins (% of proteome)	Highly Ordered, number of proteins (% of proteome)	Moderately Disordered or Moderately Flexible, number of proteins (% of proteome)	Moderately Ordered or Mildly Flexible, number of proteins (% of proteome)
Human Protein Atlas (HPA)	7970 (62.17%)	19 (0.15%)	4371 (34.1%)	459 (3.58%)
RetNet	187 (56.5%)	0 (0.0%)	125 (37.76%)	19 (5.74%)
Age-related macular degeneration (AMD)	70 (44.3%)	0 (0.0%)	76 (48.1%)	12 (7.59%)
Glaucoma	14 (43.75%)	0 (0.0%)	17 (53.12%)	1 (3.12%)
Diabetic retinopathy (DR) without gliosis (DR(−)G) proteome	77 (44.77%)	0 (0.0%)	88 (51.16%)	7 (4.07%)
Diabetic retinopathy with gliosis (DR( +)G) proteome	48 (46.6%)	0 (0.0%)	55 (53.4%)	0 (0.0%)

**Table 3:** Protein counts for those overlapping with the human protein atlas (HPA) and proteins unique to disease-specific retinal proteomes and the RetNet protein set

Protein Group	Overlap with HPA	Unique to HPA
RetNet	290	41
Age-related macular degeneration (AMD)	110	48
Glaucoma	22	10
Diabetic retinopathy (DR) without gliosis (DR(−)G) proteome	153	19
Diabetic retinopathy with gliosis (DR( +)G) proteome	97	6

## Data Availability

Data is provided within the manuscript or supplementary information files.

## Abbreviations

IDP: Intrinsically disordered proteins
IDR: Intrinsically disordered region
LLPS: Liquid–liquid-phase separation
HPA: Human Protein Atlas
AMD: Age-related macular degeneration
DR: Diabetic retinopathy
DR(+ G): Diabetic retinopathy with gliosis
DR(-G): Diabetic retinopathy without gliosis
RIDAO: Rapid intrinsic disorder analysis online
PONDR®: Predictors of naturally disordered regions
ADS: Average disorder scores
PPDR: Percentages of predicted disordered residues
ANOVA: Analysis of variance
DOF: Degrees of freedom
CH: Charge-hydropathy
CDF: Cumulative distribution function
Q1: Quadrant 1
Q2: Quadrant 2
Q3: Quadrant 3
Q4: Quadrant 4
PSP: Phase separating protein
GBDT: Gradient boosting decision tree
HSD: Honestly significant difference
PS IDRs: Phase separating intrinsically disordered regions
AUC: Area under the curve
UPR: Unfolded protein response
IRD: Inherited retinal disease
