# Supplementary material for: The effects of retinal disease on intrinsic protein disorder and liquid–liquid‑phase separation
Source: J Proteins Proteom. Author manuscript; Available in PMC 2025 Aug 11. (PMC12337953; doi:10.1007/s42485-025-00188-6)

**Supplementary Materials**

**Title:** Quantifying the effects of retinal disease on protein intrinsic disorder and liquid-liquid phase separation

**Authors:** Nedym Hadzijahic^1^, Colin K. Kim, BS^1^, Mak B. Djulbegovic, MD MSc^2^, Michael Antonietti, BS^1^, David J. Taylor Gonzalez, MD^3^, Vladimir N. Uversky, PhD DSc^4^, Jose Pulido MD^2^, Carol L. Karp, MD^1^

**Affiliations:** ^1^Bascom Palmer Eye Institute, University of Miami, Miami, FL; ^2^Wills Eye Hospital, Thomas Jefferson University, Philadelphia, PA, USA; ^3^Hamilton Eye Institute, University of Tennessee Science Center, Memphis, TN; ^4^Department of Molecular Medicine and USF Health Byrd Alzheimer’s Research Institute, Morsani College of Medicine, University of South Florida, Tampa, FL

**Correspondence:**

**Name:** Carol L. Karp, MD

**Address:** 900 NW 17^th^ Street, Miami, FL 33136

**Telephone:** 305-326-6156

**Email:** [ckarp@med.miami.edu](mailto:ckarp@med.miami.edu)

**Grant Support:** The work enclosed in this article received no external funding.

**Abbreviations**: IDP (intrinsically disordered proteins), IDR (intrinsically disordered region), LLPS (liquid-liquid phase separation), Human Protein Atlas (HPA), AMD (age-related macular degeneration), DR (diabetic retinopathy), DR(+)G (diabetic retinopathy with gliosis), DR(-)G (diabetic retinopathy without gliosis), RIDAO (Rapid Intrinsic Disorder Analysis Online), PONDR® (Predictors of Naturally Disordered Regions), ADS (Average Disorder Scores), PPDR (Percentages of Predicted Disordered Residues), ANOVA (analysis of variance), DOF (degrees of freedom), CH (charge-hydropathy), CDF (cumulative distribution function), Q1 (Quadrant 1), Q2 (Quadrant 2), Q3 (Quadrant 3), Q4 (Quadrant 4), phase separating protein (PSP), Gradient Boosting Decision Tree (GBDT), HSD (Honestly Significant Difference), phase separating intrinsically disordered regions (PS IDRs), area under the curve (AUC), unfolded protein response (UPR), inherited retinal disease (IRD)


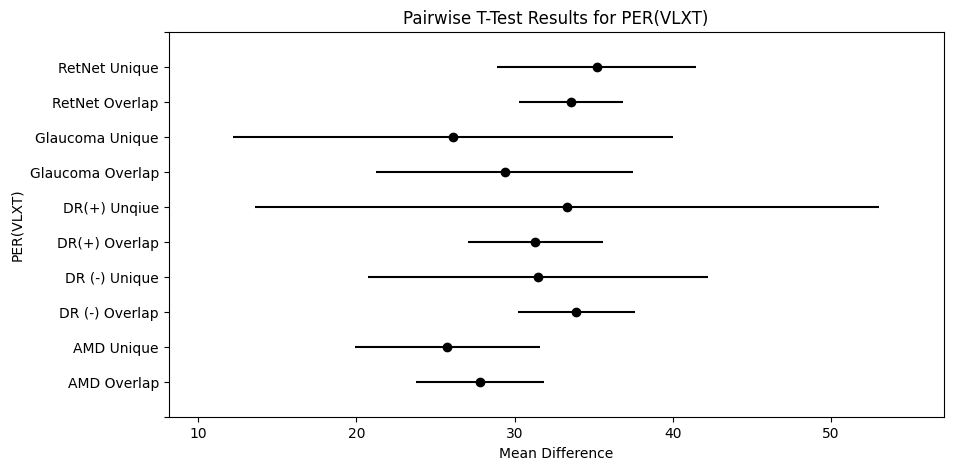


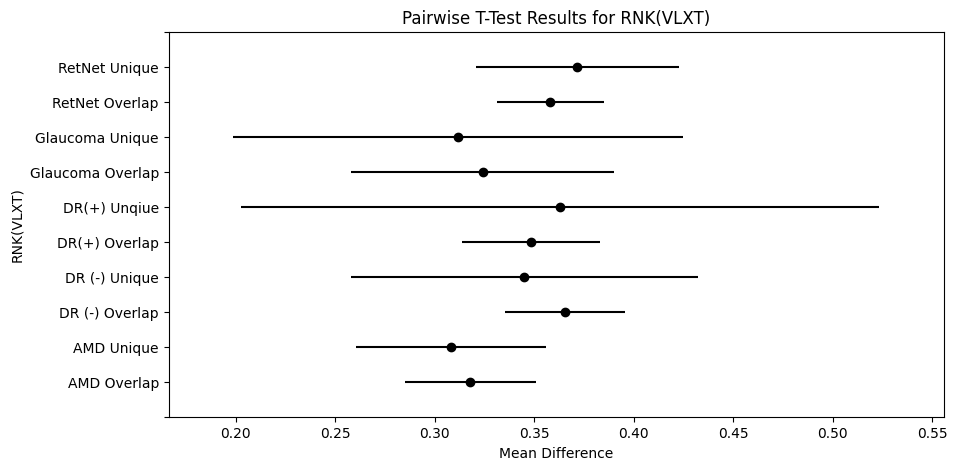


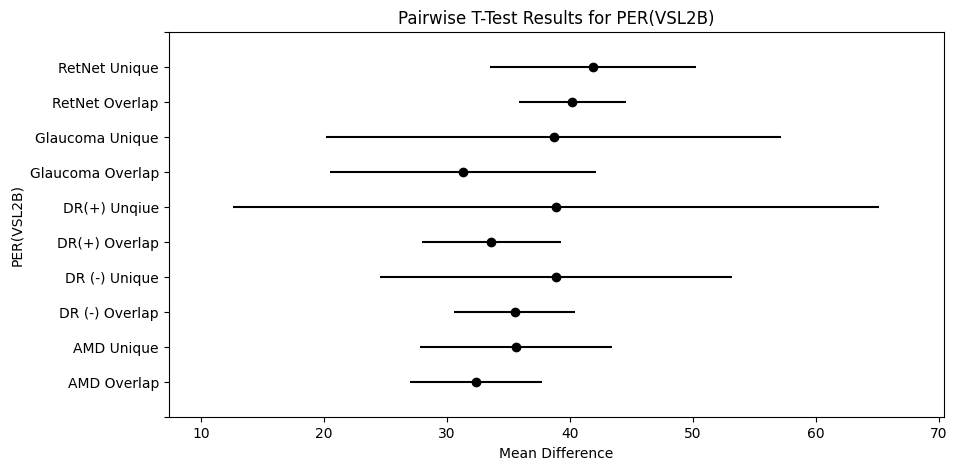


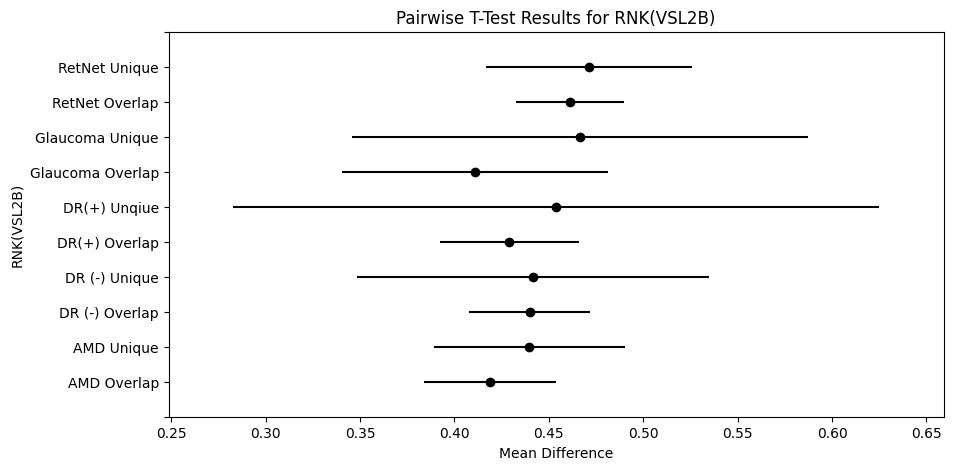


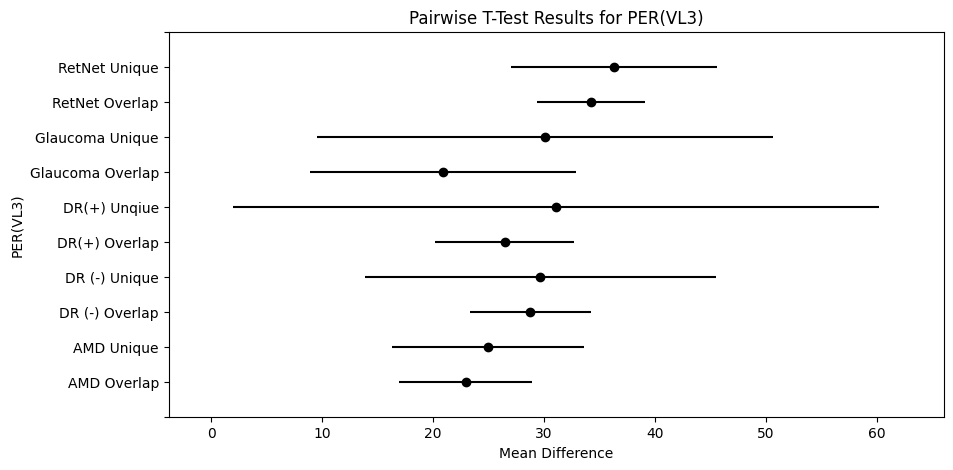


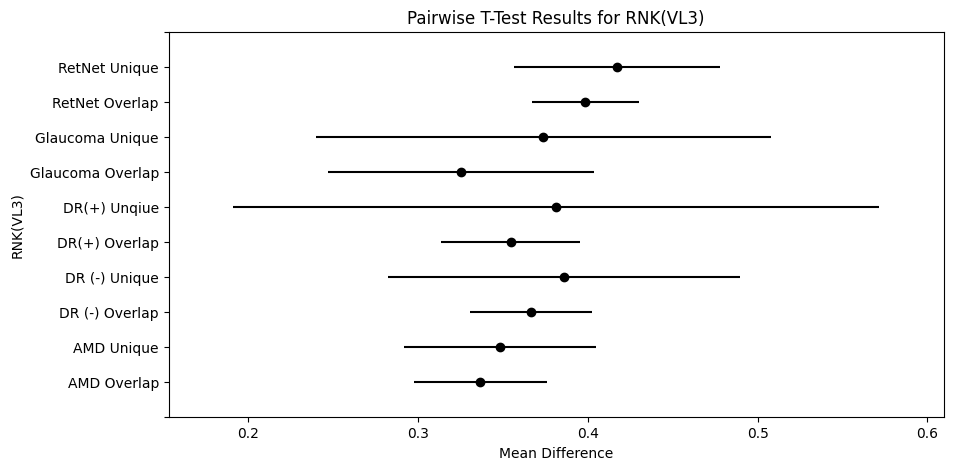


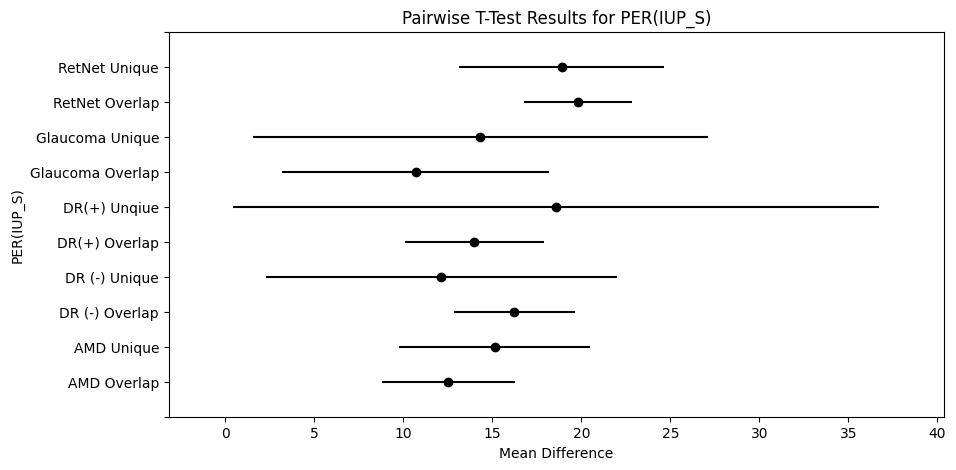


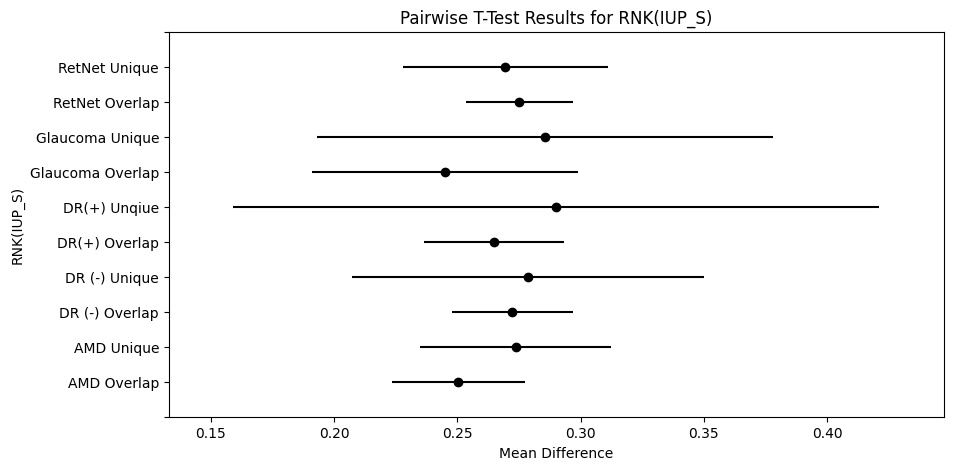


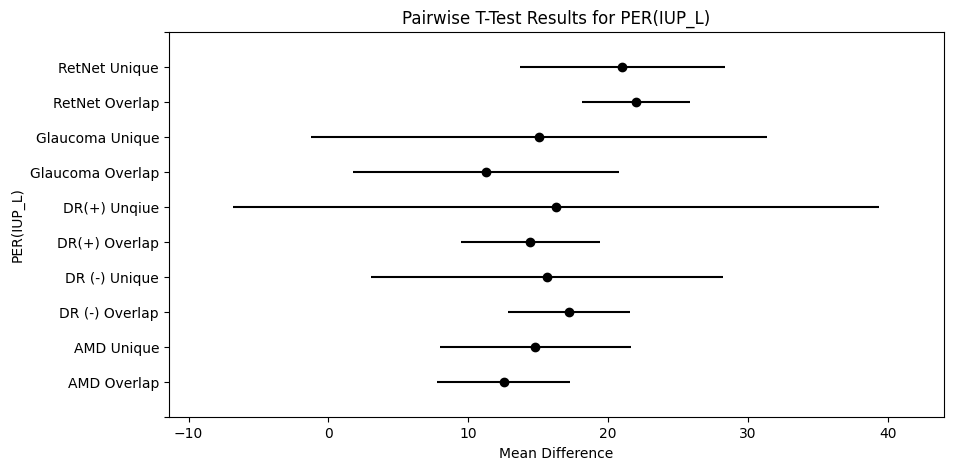


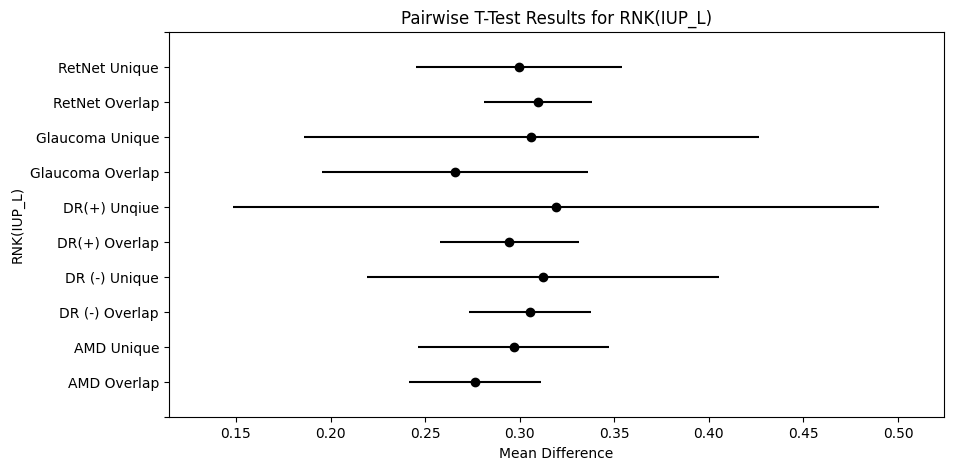


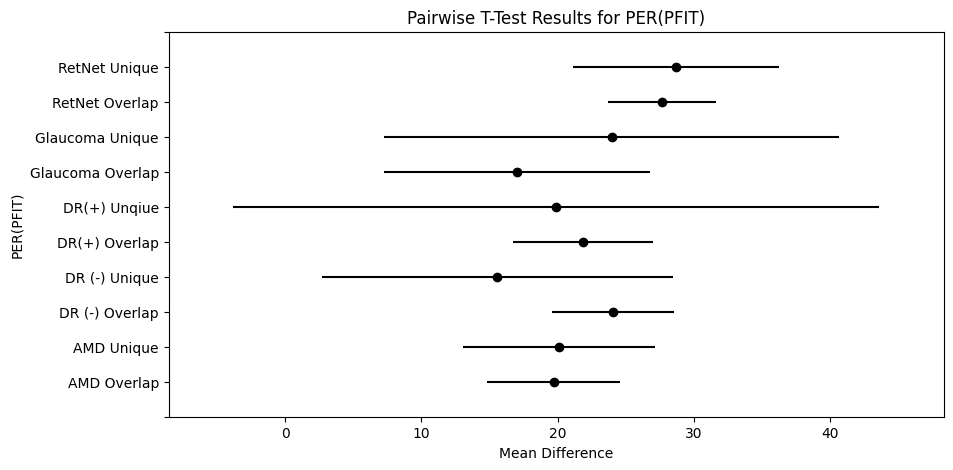


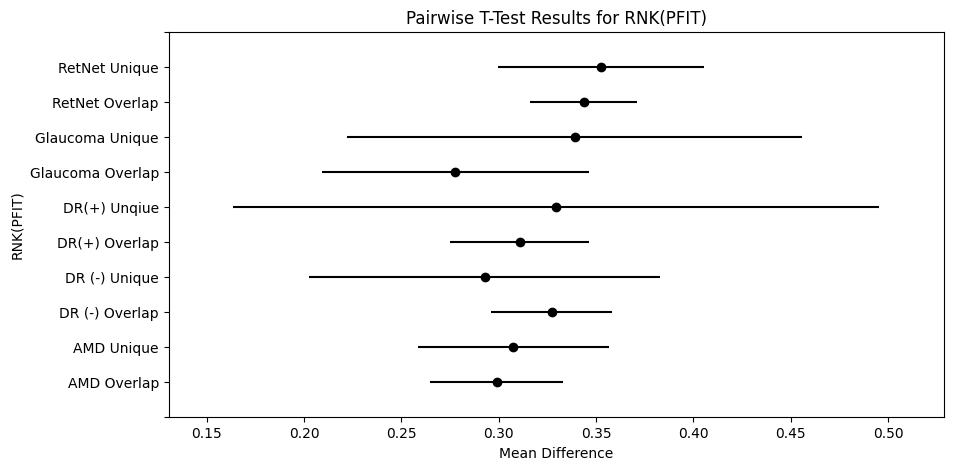


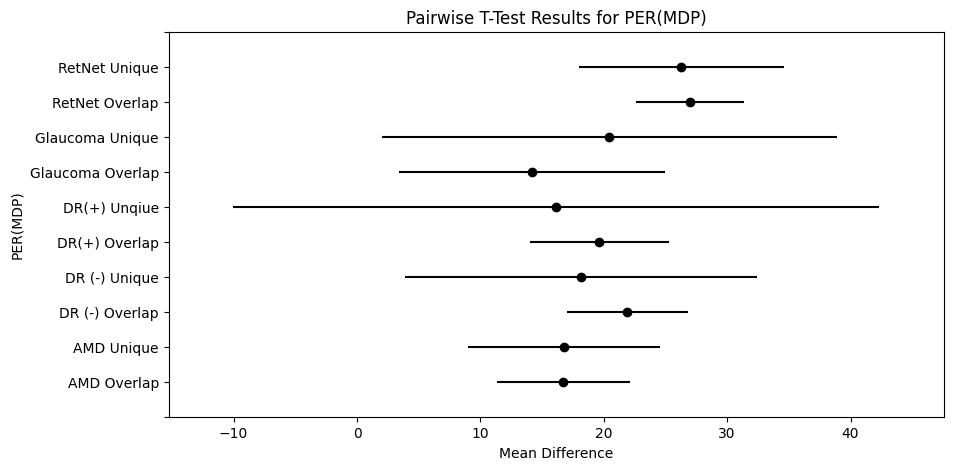


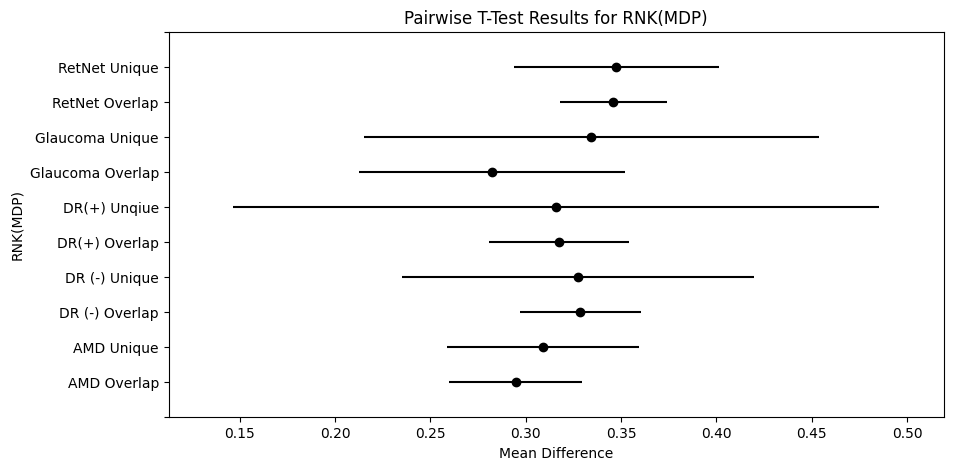


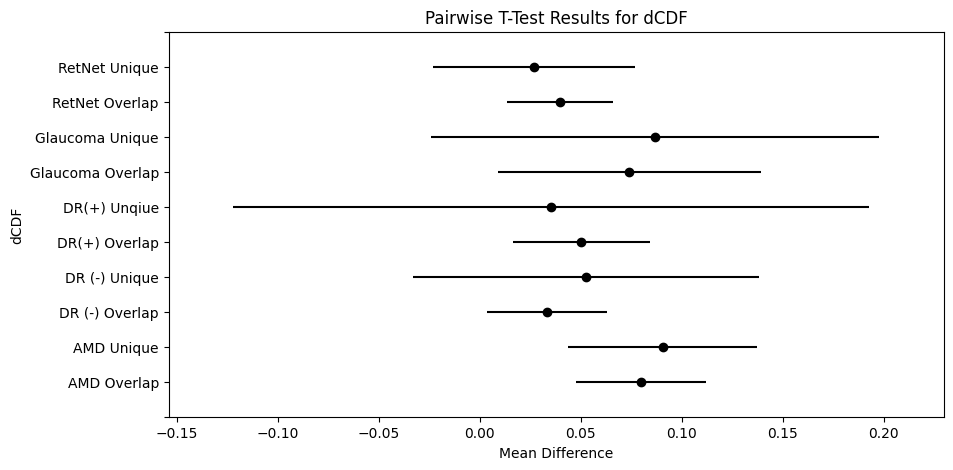


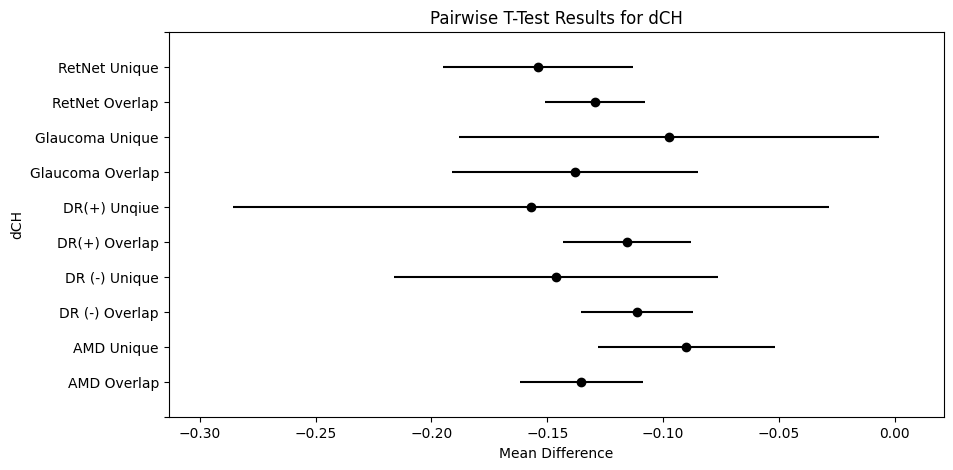


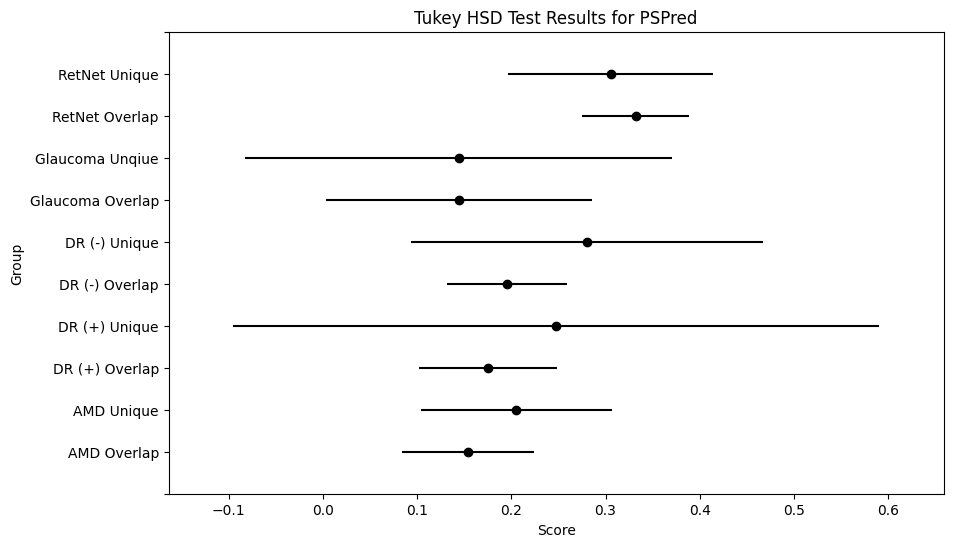

Supplement: Supplemental 1 [file NIHMS2097590-supplement-Supplemental_1.docx]
